# Supplementary material for: Statistical learning dynamically shapes auditory perception
Source: NPJ Sci Learn. 2025 Jun 19;10:41. doi: 10.1038/s41539-025-00328-z (PMC12179262; doi:10.1038/s41539-025-00328-z)
Supplement: Supplementary file 1 — Supplementary information [file 41539_2025_328_MOESM1_ESM.pdf]

Supplementary Information

Statistical learning dynamically shapes auditory perception

Sahil Luthra[1], Austin Luor[1], Adam T. Tierney, Frederic Dick, Lori L. Holt

Corresponding author: Lori L. Holt
Email: lori.holt@austin.utexas.edu

[1] equal contributions

This file includes:

Supplementary Table 1
Supplementary Table 2
Supplementary Table 3
Supplementary Figure 1
Supplementary Figure 2

Supplementary Table 1. Participant Demographics

Note that age and gender reflect only those participants included in analyses. For the tone-in-noise detection task, exclusion was by participant and not by trial. Exclusion criteria for the tone-in-noise detection task included: (1) very high tone-in-noise thresholds (-14.5 dB SNR or higher) consistent with extremely poor performance and the possibility of hearing loss, inattention or other challenges to participation; (2) individuals who reported not hearing the continuous white noise masker for the entirety of the experiment; (3) participants with very low thresholds (-26.5 dB SNR or lower) that led us to suspect errors in masking noise presentation. Exclusion criteria for the duration decision task included: (1) performance on no-feedback trials that was at or below chance, on average. At-chance performance was determined based on permutation testing with 10,000 permutations and an alpha level of 0.05. ***Table 2*** *indicates at-chance performance levels for each duration decision experiment. In addition, incorrect decisions and decisions that were made fewer than 300 ms after stimulus onset or 1500 ms after stimulus onset were excluded on a by-trial basis.*

Experiment	Age (SD)	# Participants (Gender)	# Excluded Participants	% Trial Loss (Duration Decision Only)
**Exp 1a**	24.5 (3.8)	N = 28 (5 female, 22 male, 1 non-binary)	2	—
**Exp 1b**	24.7 (4.0)	N = 28 (12 female, 16 male)	2	—
**Exp 1c**	26.7 (5.2)	N = 29 (16 female, 13 male)	1	—
**Exp 1d**	25.5 (3.7)	N = 27 (13 female, 14 male)	3	—
**Exp 1e**	24.4 (4.1)	N = 28 (12 female, 16 male)	4	—
**Exp 1f**	24.8 (3.9)	N = 30 (13 female, 16 male, 1 non-binary)	0	—
**Exp 2a**	23.7 (4.2)	N = 30 (10 female, 20 male)	0	28.11%
**Exp 2b**	23.4 (3.7)	N = 30 (13 female, 17 male)	0	25.48%
**Exp 2c**	25.4 (4.6)	N = 29 (10 female, 19 male)	1	25.16%
**Exp 2d**	25.2 (4.4)	N = 27 (11 female, 16 male)	3	23.84%
**Exp 2e**	25.4 (5.5)	N = 30 (7 female, 23 male)	0	21.97%
**Exp 3a**	24.0 (4.0)	N = 28 (14 female, 14 male)	3	—

**Exp 3b**	23.4<br>(3.6)	N = 30<br>(16 female, 14 male)	1	—
**Exp 3c**	24.4<br>(3.7)	N = 28<br>(12 female, 16 male)	2	—
**Exp 4a**	23.4<br>(3.6)	N = 31<br>(11 female, 19 male, 1 non-binary)	0	15.32%
**Exp 4b**	24.7<br>(4.6)	N = 29<br>(8 female, 21 male)	0	14.61%
**Exp 4c**	24.6<br>(4.8)	N = 27<br>(10 female, 17 male)	3	15.39%
**Exp 5a**	26.0<br>(5.7)	N = 27<br>(13 female, 14 male)	3	—
**Exp 5b**	24.9<br>(3.7)	N = 30<br>(12 female, 18 male)	0	—
**Exp 6a**	24.3<br>(4.1)	N = 28<br>(9 female, 17 male, 1 non-binary)	2	25.31%
**Exp 6b**	24.5<br>(4.1)	N = 29<br>(10 female, 18 male, 1 non-binary)	1	22.30%
**Exp 7a**	26.7<br>(4.5)	N = 30<br>(11 female, 17 male, 2 non-binary)	0	—
**Exp 7b**	26.0<br>(4.4)	N = 30<br>(7 female, 23 male)	1	—
**Exp 7c**	25.4<br>(4.3)	N = 30<br>(5 female, 25 male)	2	—
**Exp 7d**	24.3<br>(3.9)	N = 30<br>(10 female, 19 male, 1 non-binary)	2	—
**Exp 7e**	24.9<br>(4.8)	N = 29<br>(7 female, 22 male)	2	—
**Exp 7f**	24.0<br>(4.1)	N = 30<br>(9 female, 21 male)	0	—
**Exp 8a**	24.7<br>(4.3)	N = 60<br>(19 female, 39 male, 1 non-binary,<br>1 prefer not to answer)	3	—
**Exp 8b**	25.8<br>(4.6)	N = 60<br>(18 female, 41 male, 1 non-binary)	5	—

Supplementary Table 2. Summary of Experiment Protocols

Detailed protocol for each experiment, according to task, tone frequencies, trials, trials/block, and the number of statistical distribution changes. Chance performance for the duration decision task was determined based on permutation testing with 10,000 permutations and an alpha level of 0.05.

Exp	Most probable frequency	# Trials	Trials / Block	When do statistics change?	Chance-level performance (duration decision)
**Exp 1a** (Tone-in-noise detection)	1000 Hz (100% of trials)	320	32	n/a	n/a
**Exp 1b** (Tone-in-noise detection)	920 Hz (75% of trials)	320	32	n/a	n/a
**Exp 1c** (Tone-in-noise detection)	1000 Hz (75% of trials)	320	32	n/a	n/a
**Exp 1d** (Tone-in-noise detection)	1080 Hz (75% of trials)	320	32	n/a	n/a
**Exp 1e** (Tone-in-noise detection)	800 Hz and 1200 Hz (40.625% of trials each)	320	32	n/a	n/a
**Exp 1f** (Tone-in-noise detection)	800 Hz and 1200 Hz (40.625% of trials each)	320	32	n/a	n/a
**Exp 2a** (Duration decision)	920 Hz (80% of trials)	400	40	n/a	54.0%
**Exp 2b** (Duration decision)	1000 Hz (80% of trials)	400	40	n/a	54.0%
**Exp c** (Duration decision)	1080 Hz (80% of trials)	400	40	n/a	54.0%

**Exp 2d** (Duration decision)	800 Hz and 1200 Hz (40.625% of trials each)	320	32	n/a	54.7%
**Exp 2e** (Duration decision)	1st half: all frequencies equiprobable 2nd half: 800 Hz and 1200 Hz (42.5% of trials each)	640	40	After 8 blocks	53.3%
**Exp 3a** (Tone-in-noise detection)	1000 Hz (75% of trials)	320	32	n/a	n/a
**Exp 3b** (Tone-in-noise detection)	1155 Hz (75% of trials)	320	32	n/a	n/a
**Exp 3c** (Tone-in-noise detection)	1000 or 1155 Hz, switching throughout experiment (75% of trials)	640	32	After every 5 blocks	n/a
**Exp 4a** (Duration decision)	1000 Hz (80% of trials)	320	32	n/a	54.7%
**Exp 4b** (Duration decision)	1155 Hz (80% of trials)	320	32	n/a	54.7%
**Exp 4c** (Duration decision)	1000 or 1155 Hz, switching throughout experiment (75% of trials)	640	32	After every 5 blocks	53.3%
**Exp 5a** (Tone-in-noise detection)	1st half: all frequencies equiprobable 2nd half: 1000 Hz (80%)	640	40	After 8 blocks	n/a
**Exp 5b** (Tone-in-noise detection)	1st half: 1000 Hz (80%) 2nd half: all frequencies equiprobable	640	40	After 8 blocks	n/a
**Exp 6a** (Duration decision)	1st half: all frequencies equiprobable 2nd half: 1000 Hz (80%)	640	40	After 8 blocks	53.3%

**Exp 6b** (Duration decision)	1st half: 1000 Hz (80%) 2nd half: all frequencies equiprobable	640	40	After 8 blocks	53.3%
**Exp 7a** (Tone-in-noise detection)	1st half: all frequencies (857, 905, 949, 1000, 1065, 1123, 1178 Hz) equiprobable 2nd half: 1000 Hz (71.4%)	672	42	After 8 blocks	n/a
**Exp 7b** (Tone-in-noise detection)	1st half: 1000 Hz (71.4%) 2nd half: all frequencies (857, 905, 949, 1000, 1065, 1123, 1178 Hz) equiprobable	672	42	After 8 blocks	n/a
**Exp 7c** (Tone-in-noise detection)	1st half: all frequencies (768, 857, 949, 1000, 1065, 1178, 1326 Hz) equiprobable 2nd half: 1000 Hz (71.4%)	672	42	After 8 blocks	n/a
**Exp 7d** (Tone-in-noise detection)	1st half: 1000 Hz (71.4%) 2nd half: all frequencies (768, 857, 949, 1000, 1065, 1178, 1326 Hz) equiprobable	672	42	After 8 blocks	n/a
**Exp 7e** (Tone-in-noise detection)	1st half: all frequencies (732, 809, 905, 1000, 1123, 1244, 1410 Hz) equiprobable 2nd half: 1000 Hz (71.4%)	672	42	After 8 blocks	n/a
**Exp 7f** (Tone-in-noise detection)	1st half: 1000 Hz (71.4%) 2nd half: all frequencies (732, 809, 905, 1000, 1123, 1244, 1410 Hz) equiprobable	672	42	After 8 blocks	n/a
**Exp 8a** (Tone-in-noise detection)	1st half: 20 frequencies (range: 800-1200 Hz, 37-cent steps), all equiprobable (5% each) 2nd half: 1000 Hz (100% of trials)	640	40	After 8 blocks	n/a

**Exp 8b** (Tone-in-noise detection)	1st half: 1000 Hz (100% of trials) 2nd half: 20 frequencies (range: 800-1200 Hz, 37-cent steps), all equiprobable (5% each)	640	40	After 8 blocks	n/a

Supplementary Table 3. Detailed Analysis Information.

	A	B	C	D	E	F	G	H
1	**Experiment**	**Condition(s)**	**Result text**	**Test in JASP**	**Experimental condition filter**	**JASP-file**	**Additional info**	**Excluded Participants**
2	1	b-f	Frequency probability In Exp 1, stimulus probability strongly modulates tone detection in noise across Exp 1b-f with better detection of high-probability frequencies at the distribution mode (Fig 2a; ANOVA, Freq x Exp interaction, $p = 1.761 \times 10^{-31}$, eta-sq = 0.169). strongly modulates tone detection in noise across Exp 1b-f	Frequency x Exp interaction	none	Exp1a-f-TPS-AccSummary240508.jasp		
3	1	1a, 1b-d	Detection of only 1000 Hz (Exp 1a: 100% probability; average accuracy 77.9%) does not differ from detection of the highest-probability frequency in unimodal distributions (Exp 1b-d: 75% probability; average accuracy 75.3%; ANOVA, p = 0.242, eta-sq = 0.012).	ANOVA	exclude ProbabilityOfFrequency '40' (TPS dual conditions, e-f)	Exp1a-f-MostProbableFreqAcc.jasp		
4	1	1b-d, 1e-f, 1a	But detection of the 40%-probable frequencies in bimodal distributions is lower than when a single frequency is 80% or 100% probable (Exp 1e-f: 40.6% probability; average accuracy 70.3%; ANOVA, p = 0.006, eta-sq = 0.053 versus Exp 1b-d, ANOVA, p = 0.003, eta-sq = 0.101, versus Exp 1a).	ANOVA	exclude ProbabilityOfFrequency '100' (TPS 1000) OR '80' (all others)	Exp1a-f-MostProbableFreqAcc.jasp		
5	1	1b-d	Proximity to the high probability tone also influences detection (**Fig 3a**). The low-probability frequencies of Exp 1b-d share the same probability, yet those closer to a high-probability frequency are better detected than those further away (ANOVA, p = 0.014, eta-sq = 0.022).	RM ANOVA, Main effect of DistFromProbFreq	none	Exp1b-c-d-TPS-byDistFromHighProbFreq-240508.jasp		
6	1	1c	When the high-probability frequency is centered in the range of frequencies defining the distribution, this graded detection accuracy difference is symmetric (far < near to high-probability frequency, ANOVA, p = 0.004, eta-sq = 0.259).	RM ANOVA, Main effect of DistFromProbFreq	DistanceFromHighProbabilityFrequency,FilterByExpt, Filter out b and d	Exp1b-c-d-TPS-byDistFromHighProbFreq-240508.jasp		
7	1	1b, 1d	When the high-probability frequency is nearer to the distribution edge (Exp 1b and Exp 1d), there is an asymmetric detection curve (ANOVA, p = 0.015, eta-sq = 0.034): a sharp detection decrement toward the distribution edge is contrasted with a more gradual 'ski slope' decrement toward the middle of the frequency range.	Main effect, DistAccordingToPosInRange	none	Exp1b-d-TPS-EdgeMiddleNearMidFar.jasp	Edge frequency accuracy is < Middle (p = 0.016), and Far < Middle (p = 0.038).	
8	1	1e	More complex probability distributions also modulate detection (Fig 2b). Exp 1e shows that a bimodal probability distribution with higher-probability (40.6%) frequencies at the edges of the distribution (800 and 1200 Hz) induces a 'dual spotlight' across the frequency dimension. Listeners detect the higher-probability tones more accurately than neighboring low-probability tones (920 and 1080 Hz, linear contrast, Bonferroni-corrected (920 and 1080 Hz, $p = 3.590 \times 10^{-5}$, Cohen's d = 0.757) as well as the middle 1000 Hz tone (linear contrast as above, p = 0.072, Cohen's d = 0.418).	SingleConditionANOVA Exp1e OR f With contrasts, Freq effect; linear contrasts 1 and 2, Bonferroni corrected (e.g., p-values x # of contrasts = 3)	filter out 'f'	Exp1e-f-TPS-AccSummary240508.jasp		
9	1	1f	Exp 1f falsifies this hypothesis. Changing the initial threshold-setting frequency to 1080 Hz elicits a similar "W" profile and, importantly, replicates the overall 'dual spotlight' at 800 and 1200 Hz (linear contrast as above, $p = 4.62 \times 10^{-8}$, Cohen's d = 0.985, **Fig 3b**).	SingleConditionANOVA Exp1e OR f With contrasts, Freq effect; linear contrast 1, Bonferroni corrected (e.g., p-values x # of contrasts = 3)	filter out 'e'	Exp1e-f-TPS-AccSummary240508.jasp	Reported p-value is for the Bonferroni-corrected contrast of 800 & 1200 vs 920 & 1080 (contrast 1)	
10	2	2a-c	Across Exp 2a-c, the probability of a tone's *frequency* significantl impacts the speed of duration decisions ($p = 7.620 \times 10^{-7}$, eta-sq=0.017, **Fig 3a**).	Main effect of frequency, RM ANOVA	none	Exp2abc-AllConds-FINAL-RT-Filt300-1500ms-FreqXDur.jasp		Excluded 1 subject: 5304786 from 1080Bias
11	2	2a-c	Response times (RTs) vary are faster for tones with high, compared to low, probability frequencies (linear contrast, unpooled error, $p = 1.628 \times 10^{-17}$, Cohen's d = 0.516).	Linear contrast of HighPro vs Far+Near	none	Exp2a-c-PSID-RT-FreqDistXDuration_300-1500ms.jasp		Excluded 1 subject: 5304786 from 1080Bias
12	2	2a-c	Compared to RTs to the most probable frequency, those to the adjacent low-probability frequencies are slower (Holm-corrected posthoc comparison, $p = 4.316 \times 10^{-12}$, Cohen's d = 0.387) and frequencies furthest away from the high-probability frequency are yet slower than the frequencies nearer the high-probability one (Holm-corrected $p = 5.137 \times 10^{-6}$, Cohen's d = 0.258)	Post-hoc comparisons table, Holm-corrected	none	Exp2a-c-PSID-RT-FreqDistXDuration_300-1500ms.jasp	Holm's corrected posthoc table	Excluded 1 subject: 5304786 from 1080Bias
13	2	2a-c	(These patterns hold true for each Exp 2a-c study, p < .05 Holm-corrected).	Holm-corrected ANOVAs	filter by each condition separately	Exp2a-c-PSID-RT-FreqDistXDuration_300-1500ms.jasp	Holm's corrected posthoc table	Excluded 1 subject: 5304786 from 1080Bias
14	2	2d	However, unlike the dual spotlight for tone detection in Exp 1e-f, there is no significant RT advantage for making duration decisions about the higher-probability 800 and 1200 Hz tones in Exp 2d (Fig 3b; ANOVA effect of frequency, p = 0.526, eta-sq = 0.010)	RM-ANOVA, main effect of frequency	none	Exp2d-PSID-Bimodal-RT-FreqXDuration.jasp		Excluded 3 subjects: 6492029, 6492881, 6491877
15	2	2e	Indeed, and unexpectedly, when tone frequencies are equiprobable in the first half of trials, decision RTs are longer for 800 Hz and 1200 Hz compared to other frequencies (p = 0.031, eta-sq = 0.026; posthoc linear contrast p = 0.002, Cohen's d = 0.205).	RM-Anova on durations, equiprobable only;	none	Exp2e-PSID-EquiToBimodal-RT-FreqXDuration.jasp	note in general that Greenhouse-Geisser always used	
16	2	2e	Investigating this, we observe a novel frequency-duration perceptual bias: duration decisions for lower-frequency tones (800, 920 Hz) are more accurate and faster for long (90 ms) compared to short (50 ms) tones whereas those for the highest frequency tone (1200 Hz) are more accurate and faster for short compared to long tones (Fig S1; Frequency x Duration interaction, RT: p = 0.003, eta-sq = 0.029	RM-ANOVA, interaction of frequency and duration "RM-ANOVA on RTs with duration as factor"	none	Exp2e-PSID-EquiToBimodal-RT-FreqXDuration.jasp		
17	2	2e	….Accuracy (Acc): p = 3.738 x 10-5, eta-sq = 0.037)	Repeated Measures ANOVA, Frequency x Duration interaction	none	Exp2e-PSID-EquiToBimodal-Acc-FreqXDuration.jasp		
18	2	2d	This longer-lower/shorter-higher bias is mirrored, but only qualitatively, in Exp 2d (Fig S1; p > 0.05, eta-sq = 0.010)	Repeated Measures ANOVA, Frequency x Duration interaction	none	Exp2d-PSID-Bimodal-RT-FreqXDuration.jasp		Excluded 3 subjects: 6492029, 6492881, 6491877
19	2	2a-c	When we inspect the data from Exp 2a-c (Fig S1), we also observe the longer-lower/shorter-higher bias in the context of the unimodal distributions (ANOVA, Frequency x Duration interaction, RT: p = 3.968 x 10-6, eta-sq = 0.020	Repeated Measures ANOVA, Frequency x Duration interaction	none	Expt2abc-AllConds-FINAL-RT-Filt300-1500ms-FreqXDur.jasp		Excluded 1 subject: 5304786 from 1080Bias
20	2	2a-c	Acc: p = 0.003, eta-sq = 0.020).	Repeated Measures ANOVA, Frequency x Duration interaction	none	Exp2a-c-PSID-Acc-FreqXDuration.jasp		Excluded 1 subject: 5304786 from 1080Bias
21	3	3a-b	Across Exp 3a and Exp 3b, we find an equal and opposite effect of frequency probability, with the more-probable tone detected on average ~6% more accurately than the low probability tone (Fig 5; ANOVA, Frequency x Probability interaction, p = 3.361 x 10-6 eta-sq = 0.073).	Repeated Measures ANOVA, Frequency x Experiment interaction	none	Exp3a-b-TPS-AccSummary240508.jasp		

	A	B	C	D	E	F	G	H
22	4	4a-b	In Exp 4a and Exp 4b, RTs to the high probability tone frequency are ~28 ms faster, on average, than those to the low-probability frequency (ANOVA. p = 1.375 x 10-6 , eta-sq = 0.010)	Repeated Measures ANOVA, Frequency x FreqProbability interaction	none	Exp4a-b-PSID-RT-FreqXDur.jasp		
23	4	4a-b	We also observe the perceptual bias of Exp 2 even in this impoverished probability distribution, with faster RTs for long-low/short-high duration-to-frequency pairings (Fig S1; ANOVA, Frequency x Duration interaction, RT: p = 9.34 x 10-6, eta-sq = 0.013	Repeated Measures ANOVA, Frequency x Duration interaction	none	Exp4a-b-PSID-RT-FreqXDur.jasp		
24	4	4a-b	Acc: p = 6.318 x 10-5, eta-sq =0.023	Repeated Measures ANOVA, Frequency x Duration interaction	none	Exp4a-b-PSID-Acc-FreqXDur.jasp		
25	3	3c	In the statistically volatile context established by Exp 3c, there is a detection advantage for the more probable frequency, with significant 'flips' in detection accuracy related to short-term reversals in tone probability for the first three blocks of Exp 3c (Fig 5; ANOVA, Frequency x Block interaction, p = 2.495 x 10-5, eta-sq =0.099, each block p < 0.05). In the final block, there is no significant difference in detection accuracy across frequencies.	Repeated Measures ANOVA, Frequency x TimeByQuarter	linear contrasts for each block comparison of the two conditions, Bonferroni corrected	Exp3c-TPS-AccSummary240508.jasp		
26	4	4c	Likewise, transient changes in probability distribution affect the efficiency of perceptual decisions in Exp 4c (ANOVA, Frequency x Block interaction, p = 5.253 x 10-7, eta-sq =0.040). RTs are fastest for the more probable frequency in all blocks (all p < 0.05 Bonferonni-corrected).	RM-ANOVA, Freq x Block x Duration; Freq x Block interaction	linear contrasts for each block comparison of the two conditions, Bonferroni corrected	Exp4c-PSID-RT-FreqXDur.jasp		Excluded 3 subjects: 6259715, 6259743, 6259980
27	4	4c	Even in this dynamic context we observe the systematic frequency-duration perceptual bias discovered in Exp 2 (Fig S1; ANOVA, Frequency x Duration interaction, RT: p = 0.019, eta-sq =0.039 ; Acc: p = 0.019, eta-sq =0.077).	RM ANOVA, Freq x Block x Duration (RT), interaction Frequency x Duration; Repeated Measures ANOVA, Frequency x Duration interaction, (Acc)	none	Exp4c-PSID-Acc-FreqXDur.jasp, Exp4c-PSID-RT-FreqXDur.jasp		Excluded 3 subjects: 6259715, 6259743, 6259980
28	5	5a	With equiprobable frequencies in the first half of Exp 5a (Fig 2), detection accuracy is relatively consistent across frequency (Fig 6; overall ~65%, with unexpectedly better detection for 800 Hz, ANOVA, p = 0.009, eta-sq =0.129).	Exp5a-FirstHalfOnlyEquiprobable-MakeSureToToggleExpt; FrequencyFirstHalfOnly;		Exp5a-b-ByFreq.jasp	Filter to be only 5a (equi first)	
29	5	5a	In the second half of Exp 5a, probabilities shift to mirror Exp 1b (1000 Hz 75%; all others 6.25%). This probability shift drives differential changes in accuracy (ANOVA, p = 8.511 x 10-7, eta-sq = 0.142).	Repeated Measures ANOVA, DistFromCentre x ExptHalf interaction		Exp5a-TPS-EquiFirst-Acc-SummaryByDist.jasp		
30	5	5a	The 1000 Hz tones, which are now more probable, are better detected than they were in the first (equiprobable) half of Exp 5a (p = 0.002, ~Cohen's d = 0.648), whereas the now-less-probable frequencies nearest (p = 0.058, Cohen's d = 0.554) and furthest (p = 0.027, Cohen's d = 0.727) from 1000 Hz, which are now less probable, are more poorly detected than they were in the first half of the study (all Bonferroni-corrected linear contrasts with pooled error)	Repeated Measures ANOVA, Linear contrasts, Bonferroni-corrected	linear contrasts, Bonferroni corrected	Exp5a-TPS-EquiFirst-Acc-SummaryByDist.jasp		
31	5	5b	In Exp 5b, we reverse distribution order (**Fig 2**). With a unimodal distribution centered on 1000 Hz in the first half of Exp 5b, detection generally resembles Exp 1c (**Fig 6**), with better accuracy for high-probability 1000 Hz compared to low-probability frequencies (Bonferroni-corrected linear contrast, p = 1.023*10-7,· Cohen's d = 1.255), but with only a numerical detection advantage for frequencies nearest (920 and 1080 Hz) versus furthest (800 and 1200 Hz) from the probable center frequency (p = 0.262, Cohen's d = 0.267, all Bonferroni-corrected).	RM-ANOVA,UnimodalFirstHalfOnly	first contrast is high versu low probability, second contrast is near versus far, Bonf corrected	Exp5b-TPS-80-20First-Acc-SummaryByDist.jasp		
32	5	5b	When tone frequencies become equiprobable mid-study, again the probability shift drives differential changes in accuracy (p = 1.815 x 10^{-4}, Eta-sq = 0.056).	Repeated Measures ANOVA	DistFromCentreXExptHalf interaction	Exp5b-TPS-80-20First-Acc-SummaryByDist.jasp		
33	5	5b	Here, the influence of the unimodal distribution carries over to confer a detection advantage to the formerly-highly-probable 1000 Hz compared to formerly low-probable-frequencies (p = 1.383 x 10^{-4}, Cohen's d = 0.577),	RM-ANOVA,Equiprobable2ndHalfOnly	first contrast (center vs near/far), Bonf corrected	Exp5b-TPS-80-20First-Acc-SummaryByDist.jasp		
34	5	5b	although detection accuracy of 1000 Hz tones decreases from the first to the second study half (1.162 x 10-5, Cohen's d = 0.496).	Repeated Measures ANOVA	3rd contrast (Centre First Minus Centre Second), Bonf corrected	Exp5b-TPS-80-20First-Acc-SummaryByDist.jasp		
35	5	5b	There is not a commensurate improvement of detection for formerly low-probability tones, despite a more than 3-fold probability increase (p = 1, Bonferroni corrected).	Repeated Measures ANOVA	5th contrast, Bonf corrected	Exp5b-TPS-80-20First-Acc-SummaryByDist.jasp		
36	6	6a	In the first half of Exp 6a, duration decision RTs across equiprobable frequencies are similar (**Fig 6**, ANOVA, p = 0.163, Eta-sq = 0.018).	RM-ANOVA-6aONLYFirstHalfOnly-Toggle!	Exclude BiasFirst; main effect of frequency	Exp6a-b-RT-FreqXExptHalfXDurationFINAL.jasp		Excluded 2 subjects: 6516557, 6517370
37	6	6a	When probabilities shift to a unimodal distribution centered on 1000 Hz at mid-study, RTs drop overall (ANOVA, p = 0.011, Eta-sq = 0.061)	Exp6a-ONLY-MakeSureToToggle!;	Exclude BiasFirst; main effect of Expt Half	Exp6a-b-PSID-RT-DistXExptHalf.jasp		Excluded 2 subjects: 6516557, 6517370
38	6	6a	Although there is a numerical RT advantage for the now-probable 1000 Hz compared to more distant frequencies, this pattern does not differ significantly across experiment half (ANOVA, p = 0.245, Eta-sq = 0.005).	Exp6a-ONLY-MakeSureToToggle!;	Exclude BiasFirst; interaction of FreqDistance x ExptHalf	Exp6a-b-PSID-RT-DistXExptHalf.jasp		Excluded 2 subjects: 6516557, 6517370
39	6	6b	In the first, unimodal probability half of Exp 6b, duration decisions exhibit the "V" shape around the high-probability 1000 Hz tone also observed in Exp 2b (ANOVA, effect of frequency, p =6.847 x 10-8, eta-sq = 0.080, Fig 6).	RM-ANOVA-6aONLY-Toggle!	Exclude EquiFirst, main effect of frequency	Exp6a-b-RT-FreqXExptHalfXDurationFINAL.jasp		Excluded 1 subject: 7100964
40	6	6b	Decisions about low-probability frequencies near to 1000 Hz are only numerically slowed compared to 1000 Hz itself (this and following test Bonferroni-corrected linear contrasts, p = 0.058, Cohen's d = 0.227) but faster than to those further away from 1000 Hz (p = 0.014, Cohen's d = 0.299).	Exp6b-ONLYFirstHalfOnly-MakeSureToToggle!	ExcludeEquiFirst, contrasts 1 & 2 (FreqDistance)bonf corrected for 2 tests	Exp6a-b-PSID-RT-DistXExptHalf.jasp		Excluded 1 subject: 7100964
41	6	6b	When all frequencies become equiprobable mid-study in Exp 6b, there is a change in the degree to which frequency modulates duration decisions (ANOVA, p = 0.024, eta-sq = 0.017),	RM-ANOVA-Expt6bOnly-TOGGLE!	ExcludeEquiFirst, Frequency x ExptHalf interaction	Exp6a-b-RT-FreqXExptHalfXDurationFINAL.jasp		Excluded 1 subject: 7100964
42	6	6b	Even though 1000 Hz is now 20% probable, RTs are not significantly different than in the first experiment half when it was 80% probable (linear contrast, p = 0.720, Cohen's d = 0.025).	Exp6b-ONLY-MakeSureToToggle!	ExcludeEquiFirst, Contrast 1	Exp6a-b-PSID-RT-DistXExptHalf.jasp		Excluded 1 subject: 7100964

	A	B	C	D	E	F	G	H
43	6	6b	Like detection in Exp 5b, there is carryover from experience with the unimodal distribution in the first half of the study, such that duration decision RTs are still modulated by frequency (ANOVA, p = 8.306 x 10-5, eta-sq = 0.068).	Exp6b-ONLY2ndHalfOnly-Toggle!	ExcludeEquiFirst, Frequency main effect	Exp6a-b-RT-FreqXExptHalfXDurationFINAL.jasp		Excluded 1 subject: 7100964
44	6	6b	RTs to report decisions for 1000 Hz continue to be significantly faster than for the now-equally-probable far frequencies (p = 0.020, Cohen's d = 0.199), although not significantly faster than nearby frequencies (p = 0.668, Cohen's d = 0.054).	Exp6b-ONLY2ndHalfOnly-MakeSureToToggle!	ExcludeEquiFirst, Contrasts 2 and 1, bonferroni corrected	Exp6a-b-PSID-RT-DistXExptHalf.jasp		Excluded 1 subject: 7100964
45	6	6a-b	Finally, we again observe the duration-frequency bias established in the prior duration decision studies (**Fig S1**, ANOVA, Frequency x Duration interaction, RT: p = 1.068 x 10^{-4}; eta-sq = 0.013	Repeated Measures ANOVA	Interaction of Frequency and Duration	Exp6a-b-RT-FreqXExptHalfXDurationFINAL.jasp		Excluded 3 subjects: See above
46	6	6a-b	Finally, we again observe the duration-frequency bias established in the prior duration decision studies (Fig S1, ANOVA, Frequency x Duration interaction … Acc: p = 0.006, eta-sq = 0.021).	Repeated Measures ANOVA	Interaction of Frequency and Duration	Exp6a-b-Acc-FreqXExptHalfXDuration.jasp		Excluded 3 subjects: See above
47	7	7a-c-e	In Exp 7a,c,e, an equiprobable distribution precedes a switch to a unimodal distribution centered on 1000 Hz. Across these three studies, detection accuracy in the equiprobable first halves does not vary across frequency (ANOVA, p = 0.399, eta-sq = 0.003) nor is it modulated by the different frequency ranges across Exp 7a,c,e (ANOVA, p = 0.115, eta-sq = 0.035) and there is no interaction of frequency and range (p = 0.119, eta-sq = 0.011).	Range Expts, EquiFirstOnly, FirstHalfOnly	Main effect of DistFromCenter, Main effect of Range, interaction of frequency and range; exclude all Bias conditions	Exp7a-c-e-EquifirstXDist.jasp		
48	7	7a-c-e + 5a	Average detection accuracy across these equiprobable distributions is 64%, which does not differ from that in the 5-frequency distribution of Exp 5 (ANOVA, p = 0.219, eta-sq = 0.039).	ANOVA (note, these data are averaged across all frequences)	Main effect of Experiment	Exp7a-c-e-PlusExp5a-FirstHalfOnlyAvgFreq.jasp		
49	7	7a-c-e	The introduction of the unimodal distribution differentially effects frequency detection, depending on distance from 1000 Hz (ANOVA, p = 1.622 x 10^{-11}, eta-sq = 0.030).	RM ANOVA on Range Expts, Equiprobable first only	Interaction of ExptHalf and DistFromCenter	Exp7a-c-e-EquifirstXDist.jasp		
50	7	7a-c-e	When 1000 Hz shifts from equiprobable (14.3%) to highly probable (71.4%), there is a small but reliable *increase* in detection accuracy (ANOVA, p = 0.002, eta-sq = 0.026). It is notable that this five-fold increase in probability (and ~16-fold increase in relative probability compared to low-probability frequencies) only confers an average 3.7% detection improvement. This mild enhancement is not significantly influenced by the range of frequencies (ANOVA, p = 0.365, eta-sq = 0.005).	Range Expts, EquiFirst Only, CenterFreq Only	Main effect of Expt Half, and Interaction of ExptHalf x Range	Exp7a-c-e-EquifirstXDist.jasp		
51	7	7a-c-e	Examining the off-center frequencies that drop in probability (14.3% to 4.8%) upon introduction of a unimodal distribution, we observe a significant *decrease* in detection accuracy of 4.7% (ANOVA, p = 4.798 x 10^{-9}, eta-sq = 0.051), the magnitude of which does not differ significantly across range (ANOVA, p = 0.337, eta-sq = 0.003).	Range Expts, EquiFirstOnly, ExcludeCenterFreq	Main effect of Expt Half, and Interaction of ExptHalf x Range	Exp7a-c-e-EquifirstXDist.jasp		
52	7	7b-d-f	Turning next to Exp 7b,d,f (**Fig 2x**), what happens when initial experience with a unimodal distribution shifts mid-study to equiprobable presentation? As now expected from prior results, detection of the high-probability unimodal center frequency is considerably more accurate than detection of improbable frequencies (linear contrast, p = 5.198 x 10^{-30}, Cohen's d = 1.332).	RangeExpts,Unimodal(Bias)First,FirstHalfOnly	Contrast -FirstHalfOnlyFreq	Exp7b-d-f-RangeExpts.jasp		
53	7	7b-d-f	Detection of low-probability frequencies is modulated by proximity to the high-probability center frequency (ANOVA, p = 0.010, eta-sq = 0.016); accuracy is higher for frequencies nearest the high-probability center frequency compared those at middle (p = 0.023, Cohen's d = 0.269) or far frequencies (p = 0.023, Cohen's d = 0.274, both contrasts Holm-corrected).	RangeExpts,Unimodal(Bias)First,FirstHalfOnly,NoCenter	Main effect of 'FirstHalfOnlyFreqNoCenter'; Post Hoc comparisons, holm-correct p-values, Int vs Near, Far vs Near	Exp7b-d-f-RangeExpts.jasp		
54	7	7b-d-f	However, the relatively preserved detection accuracy for tones near the high-probability frequency compared to those is observed only in Exp 7b for the narrow range (near vs. middle, p = 0.014, Cohen's d = 0.512, near vs. far, p = 1.161 x 10^{-3}, Cohen's d = 0.697, all linear contrasts Bonferroni-corrected).	RangeExpts,Unimodal(Bias)First,FirstHalfOnly,NoCenter	Contrasts "Range x FirstHalfOnlyFreqNoCentre" 1-4, contrast 1 is Narrow range, Near - Int; contrast 4 is Narrow range, Near - far. Values are bonferroni corrected for the 4 comparisons	Exp7b-d-f-RangeExpts.jasp		
55	7	7b-d-f	The effects on detection of proximity to the high-probability 1000 Hz are modulated by the switch to an equiprobable distribution (ANOVA, p = 3.279 x 10^{-11}, eta-sq = 0.023).	RM ANOVA on Range Expts, Unimodal (Bias) First OMNIBUS	Interaction of ExptHalf and DistFromCenter	Exp7b-d-f-RangeExpts.jasp		
56	7	7b-d-f	We observe a continued, but smaller, detection advantage for the formerly high-probability center frequency compared to formerly improbable frequencies (linear contrast, p = 1.194 x 10^{-8}, Cohen's d = 0.552.)	RangeExpts,Unimodal(Bias)First,SecondHalfOnly	Contrast 1	Exp7b-d-f-RangeExpts.jasp		
57	7	7b-d-f	This change is driven by a *decrease* (difference of 7.1%, ANOVA, p = 1.137 x 10^{-12}, eta-sq = 0.085) in detection accuracy for the center frequency as it becomes 5 times less probable, as well as a smaller (difference of ~2%,ANOVA, p = 0.007, eta-sq = 0.006) *increase* in accuracy as off-center frequencies become 3 times more probable (**Fig 2x**).	RM-ANOVA, RangeExpts, Bias-First. CentreFreqOnly, AND Range Expts, Unimodal (Bias) First OMNIBUS, NoCentreFreq	Main effect of Expt Half for both	Exp7b-d-f-RangeExpts.jasp		
58	7	7b-d-f	This residual advantage does not vary significantly with distance from the center frequency (ANOVA, p = 0.213, eta-sq = 0.002) or interact with the range of frequencies presented (ANOVA, p = 0.202, eta-sq = 0.004).	RM-ANOVA, RangeExpts, Bias-First. NoCentreFreq	Interaction of ExptHalf x DistFromCenter, ExptHalf x DistFromCenter x Range	Exp7b-d-f-RangeExpts.jasp		
59	7	7a-f, unimodal halves only	Next, we ask if hysteresis is also observed in detection accuracy for 1000 Hz in a unimodal distribution *after* prolonged initial exposure to an equiprobable distribution (second half of Exp 7a,c,e) compared to when the experiment begins with a unimodal distribution (first half of Exp 7b,d,f). We find that pre-exposure to 336 trials of the flat probability distribution diminishes detection rates for the high-probability 1000 Hz tone in the subsequent unimodal distribution by 5.8% relative to when the identical unimodal distribution is encountered first (ANOVA, p = 6.394 x 10^{-4}, eta-sq = 0.064). The persistent damping effect of encountering the equiprobable distribution is not significantly affected by the range of frequencies encountered (ANOVA, p = 0.768, eta-sq = 0.003).	'RangeExpts-UnimodaHalvesCombined-CentreFreqOnly'	Main effect of Order, Main effect of Range	Exp7a-f-RangeExpts-BiasedHalvesOnlyXDistFromTargAcc.jasp		

	A	B	C	D	E	F	G	H
60	7	7a-f, unimodal halves only	Finally, we aggregate detection data for off-center frequencies across the unimodal conditions from Exp 7a,c,e (for which it was second) and Exp 7b,d,f (for which it was first) to maximize the power to detect influences of frequency range and distance from the higher-probability center frequency. Frequency range has an effect on detection in unimodal probability distributions (ANOVA, p = 0.005, eta-sq = 0.039). Specifically, a wide frequency range impairs overall off-center detection accuracy, compared to when the frequency range is narrow (post hoc Holm-corrected for following three contrasts, p = 0.006, Cohen's d = 0.471). The middle frequency range falls in-between and differs significantly from detection in wide (p = 0.037, Cohen's d = 0.354) but not narrow ranges (p = 0.429, Cohen's d = 0.118).	RangeExpts-UnimodalHalvesCombined-NoCentreFreq	Main effect of Range, condition-wise comparisons are Holm-corrected post hoc tests	Exp7a-f-RangeExpts-BiasedHalvesOnlyXDistFromTargAcc.jasp		
61	7	7a-f, unimodal halves only	Moreover, the shape of the drop-off in detection accuracy from the high-probability center frequency is significantly graded only in the narrow frequency range, between near and mid frequency bands condition (p = 0.015, Cohen's d = 0.388) with a non-significant decrease between middle and far frequencies (p = 0.316, Cohen's d = 0.229).	RangeExpts-UnimodalHalvesCombined-NoCentreFreq	Contrasts 1-4, contrast 4 is near vs middle (narrow), contrast 3 is middle vs far (narrow); p-values Bonferroni-corrected for 4 comparisons	Exp7a-f-RangeExpts-BiasedHalvesOnlyXDistFromTargAcc.jasp		
62	8	8b	We first ask whether the consistent experience with 1000 Hz in the first half of Exp 8b yields accumulating detection accuracy improvements (**Fig 8**). It does not: accuracy in the first quarter of trials is 78% (aligned with expectations from listener-specific thresholding) then decreases slightly to plateau at 75% for the remaining trials (ANOVA, p = 0.015, eta-sq = 0.068).	CenterFreqOnlyFirstHalfByBlock	Main effect of block pair	Exp8b-21Freq1kHzFirstSubsByDistAndBlockPair.jasp		
63	8, 1	8a, 1a	Similarly, neither Exp 1a (ANOVA, p = 0.210, eta-sq = 0.050) or Exp 8a (ANOVA, p = 0.451, eta-sq = 0.014) exhibit improved detection across a block of trials with only 1000 Hz tones.	Repeated Measures ANOVA (1a), CenterFreqByBlock2ndHalf (8a)	Main effect of block; main effect of block pair 2nd half	Exp1a-TPS-1kHz-ByBlock.jasp; Exp8a-21FreqEquiFirstSubsByDistAndBlockPair.jasp		
64	8	8a	There is a similar initial detection decrement of ~5% across the first quarter of the 20-equiprobable-frequency trials of Exp 8a with no further change (ANOVA, p = 9.669 x 10^{-6}, eta-sq = 0.049)	OffCenterFreqsOnlyFirstHalfByBlockPair	Main effect, FirstHalfBlockPair	Exp8a-21FreqEquiFirstSubsByDistAndBlockPair.jasp		
65	7	7a,c,e	This same pattern emerges in the initial equiprobable blocks of Exp 7a,c,e (ANOVA, p = 1.375 x 10^{-5}, eta-sq = 0.058)	Repeated Measures ANOVA	Main effect of Block	Exp7a-c-e-7FreqEquiFirstBlocksOnlyByBlock.jasp		
66	5, 7, 8	5a, 7a,c,e, 8a	Detection accuracy for equiprobable distributions that are experienced in the first half of a study does not differ over experiments (ANOVA, Exp 5a, 7a,c,e, and 8a; p = 0.387, eta-sq = 0.024).	ANOVA	Main effect of Experiment	Exp5a+Exp7a-c-e+Exp8a-7Freq+21FreqEquiFirstBlocksOnlyBySub.jasp		
67	8, 1	8a, 1a, 8b	Turning next to the nature of the hypothesized attentional filter, we first examine whether initial experience with the 20-tone equiprobable distribution in Exp 8a (which does not include 1000 Hz) impacts subsequent detection in the 1000-Hz-only block. It does not: detection of 1000 Hz in Exp 8a did not differ from either Exp 1a (Tukey-corrected p = 0.315, Cohen's d = 0.333) or the first half of Exp 8b (Tukey-corrected p = 0.837, Cohen's d = 0.104), each of which involved blocks of trials with only 1000 Hz at the beginning of the study.	ANOVA	Tukey-corrected posthocs across experiment pairs	Exp1a+Exp8a-b1000HzOnlyPlus21Freq1000HzMeanAccBySub.jasp		
68	8	8a,b	In contrast, massed exposure to 1000 Hz in the first half of Exp 8b drives a dramatic, long-lasting, and frequency-specific detection decrement for the subsequently encountered 20 equiprobable frequencies, as compared to detection across equiprobable frequencies in Exp 8a (ANOVA, interaction of Distance-from-1000-Hz x Exp, p = 2.618 x 10^{-4}, eta-sq = 0.007)	RM ANOVA, 20-freq Dist x order, no center frequency	Interaction, DistFromCenter x experiment	Exp8a-b-TPS-21Freq-AllSubsByDistFromCenter.jasp		
69	8	8a,b	Specifically, as shown in **Fig 8**, detection of frequencies at far (2 to 3.9 semitones) and intermediate (1 to 2 semitones) distances from 1000 Hz were detected much less accurately after massed experienced with 1000 Hz (Exp 8b, linear contrasts; far: p = 2.427 x 10^{-3}, Cohen's d = 0.330; intermediate: p = 9.784 x 10^{-4}, Cohen's d = 0.346), compared to equiprobable presentation at the beginning of the study (Exp 8a). This suppressive effect was rescued by proximity to the now-absent 1000 Hz in the second half of Exp 8b, with frequencies within about a semitone from 1000 Hz eliciting detection accuracies roughly on par with those from Exp 8a (p = 0.332, Cohen's d = 0.311).	RM ANOVA, 20-freq Dist x order, no center frequency	Contrasts 4, 3, & 2 Bonferroni corrected for 4 comparisons	Exp8a-b-TPS-21Freq-AllSubsByDistFromCenter.jasp		
70	8	8a,b	One might expect that any initial learning across the 1000-Hz-only distribution would be overwhelmed by the mid-study shift to the high-uncertainty 20-frequency equiprobable distribution. However, we see the opposite: across the second half of Exp 8b, there is no significant change in overall detection accuracy (ANOVA, p = 0.165, eta-sq = 0.008), nor any change across time in relative accuracy of detection across frequencies (**Fig 8**; ANOVA, p = 0.568, eta-sq = 0.006). The large advantage for detection of frequencies near 1000 Hz compared to intermediate and far frequencies persists to the final 80 trials of Exp 8b (linear contrast, p = 0.002, Cohen's d = 0.257 **Fig 8**).	NonCenterFreqsByBlockIn2ndHalf	Main effect of BlockPair2ndHalf; Interaction DistFromCenter x BlockPair2ndHalf; contrast 1 (last block pair)	Exp8b-21Freq1kHzFirstSubsByDistAndBlockPair.jasp		
71	8	8a,b	Here, there is strong suppression of frequencies far and intermediate distances from 1000 Hz in Exp 8b compared to detection of the same frequencies in the equiprobable half of Exp 8a. As with the within-experiment comparison, this difference is observed through the entirety of the second half of the study, again extending even to the last quarter of trials (linear contrast, p = 0.008, Cohen's d = 0.440).	Repeated Measures ANOVA	Contrast 1, comparison of 2 conditions in last block pair	Exp8a-b-21Freq-Cross-ExptCompareFarIntOverBlockPairs.jasp		

Supplementary Figure 1. A novel frequency-duration perceptual bias

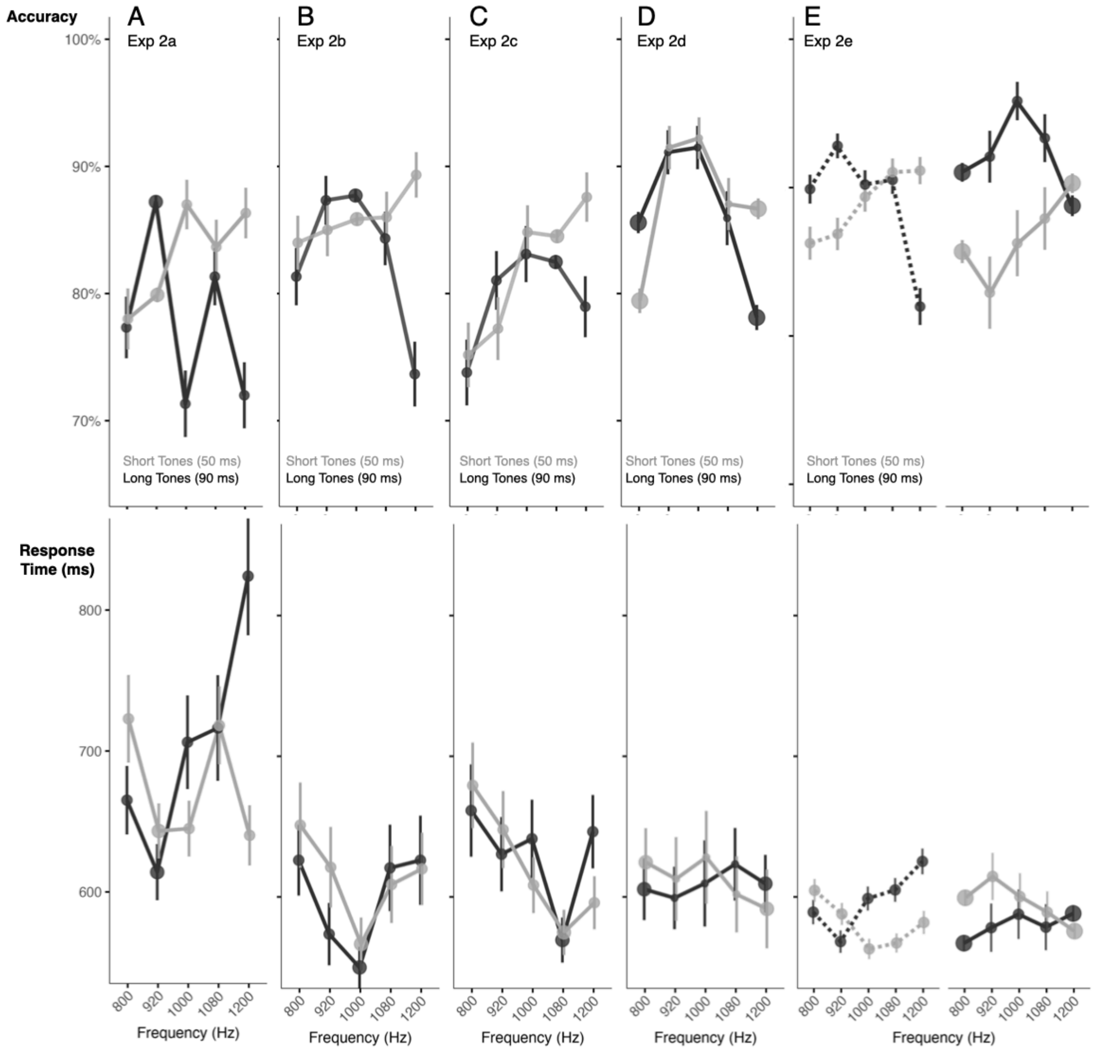

Supplementary Figure 1. A novel frequency-duration perceptual bias. Each panel plots mean duration decision accuracy as a function of acoustic frequency for Exp 2a-c, respectively (Panels A, B, C). Error bars reflect standard error of the mean. Responses to short (50 ms) tones are plotted in grey and responses to long (90 ms) tones are plotted in black. Marker size indicates tone probability. Duration decisions for lower-frequency tones (800, 920 Hz) are more accurate and faster for long (90 ms) compared to short (50 ms) whereas those for the highest tone frequency (1200 Hz) are more accurate and faster for short compared to long tones. This underlying perceptual bias is greatest at the edges of the distribution range, interacting substantially with the bimodal distributions of Exp 2d-e. This helps to explain the absence of a dual spotlight influence of bimodal distributions in Exp 2d-e that mirrors those observed for detection in Exp 1e-f.Fig. S2. Type or paste legend here. Paste figure above the legend.

Supplementary Figure 2. The influence of experience with a single frequency (point distribution) persists across the second half of the study

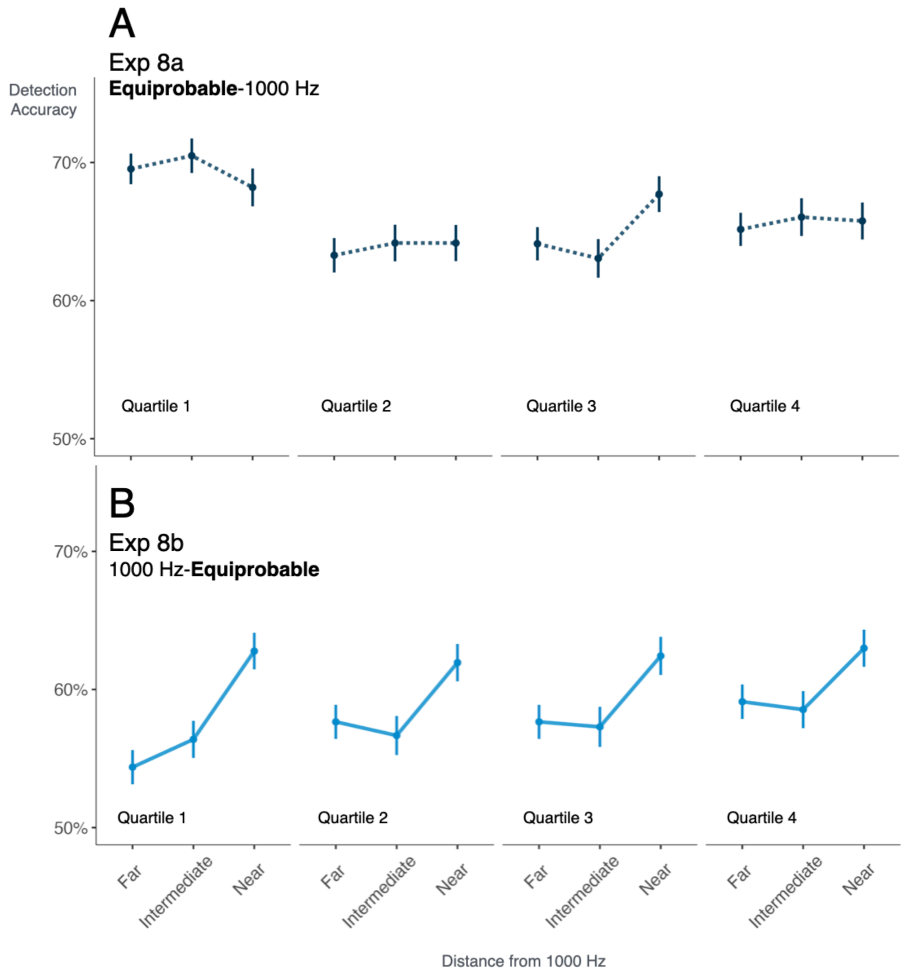

Supplementary Figure 2. The influence of experience with a single frequency (point distribution) persists across the second half of the study. Each panel plots mean detection accuracy across equiprobable distributions (first half of experiment in Exp 8a, second half of experiment in Exp 8b) as a function of distance of tone frequency from 1000 Hz (near, intermediate, far) and quartile of trials within the half of the experiment involving the equiprobable distribution. Error bars correspond to standard error of the mean. **(A)** Exp 8a provides an estimate of baseline detection accuracy across the 20 frequencies in the first half of the study. (B) In contrast, detection accuracy in Exp 8b — which follows experience with 1000 Hz only in the first half of the study — is significantly suppressed for tones with frequencies intermediate and far distances from 1000 Hz. Note that this persists even into the final quartile (80 trials) of Exp 8b. The absence of 1000 Hz in these equiprobable distributions rules out trial-wise perceptual interactions or a relative probability difference for the lingering influence of the point distribution experienced at 1000 Hz in the first half of Exp 8b.
